# Forms of Albumen in the Urine

**Published:** 1907-05

**Authors:** 


### FORMS OF ALBUMEN IN THE URINE.

Hastings and Hoobler accept the general conception of the term albuminuria, namely, albumin in the urine as excreted from the bladder. The albumin bodies of endogenous origin occurring in the urine include serum albumin, serum globulin, fibrinogen, fibrin nucleoalbumin, nucleoproteid, mucin, albumose, digestive albumose, and true peptone. The four of these which are most significant are serum albumin, serum globulin, nucleoalbumin, and albumose. Four tests were used in a great number of urine analyses made by the authors: (1) Heat and acetic acid; (2) cold acetic acid with diluted urine; (3) heat and acetic acid, after first adding saturated sodium chloride solution; (4) the test for serum globulin. Serum albumin must be differentiated from nucleoalbumin in testing urine, the latter being of little clinical importance. The cases of well established Bright's disease in which the symptoms and physical signs pointed to renal lesion included only 12.59 per cent. of all the cases in which albuminuria was found by the authors. Albuminuria does not necessarily nor usually indicate nephritis. If casts are present also there is probably nephritis, either temporary or permanent. The authors wish to emphasize the frequency of non-renal albuminuria and the occurrence of nucleoalbuminuria alone in a large percentage of cases. The separation of the albumin bodies as a matter of practice is not necessary.—Amer. Jour. Med. Science.

